# Known in the nursing home: development and evaluation of a digital person-centered artistic photo-activity intervention to promote social interaction between residents with dementia, and their formal and informal carers

**DOI:** 10.1186/s12877-021-02632-w

**Published:** 2022-01-06

**Authors:** Josephine Rose Orejana Tan, Petra Boersma, Teake P. Ettema, Laurence Aëgerter, Robbert Gobbens, Max L. Stek, Rose-Marie Dröes

**Affiliations:** 1 Department of Psychiatry, Amsterdam University Medical Centers, location VUmc /Amsterdam Public Health Research institute, Amsterdam, The Netherlands; 2 Faculty of Health, Sports and Social Work, Inholland University of Applied Sciences, Amsterdam, The Netherlands; 3 Visual artist, https://laurenceaegerter.com/; 4 Zonnehuisgroep Amstelland, Amstelveen, The Netherlands; 5 Department of Family Medicine and Population Health, Faculty of Medicine and Health Sciences, University of Antwerp, Antwerp, Belgium; 6 Department of Old Age Psychiatry, Regional Mental Health organization GGZ inGeest, Amsterdam, Netherlands

**Keywords:** Dementia, nursing homes, tablet computer, artistic intervention, photos, psychosocial intervention

## Abstract

**Background:**

To address the lack of social interaction and meaningful activities for persons with dementia (PWD) in nursing homes an artistic Photo-Activity was designed. The present study aims to develop a digital version of the Photo-Activity and to investigate its implementation and impact on nursing home residents with advanced dementia, and their (in)formal carers.

**Methods:**

First, within a user-participatory design, a digital-app version of the Photo-Activity will be developed and pilot-tested, in co-creation with (in)formal carers and PWD.

Next, the feasibility and effectiveness of the Photo-Activity versus a control activity will be explored in a randomized controlled trial with nursing home residents (*N*=90), and their (in)formal carers. Residents will be offered the Photo-Activity or the control activity by (in)formal carers during one month. Measurements will be conducted by independent assessors at baseline (T0), after one month (T1) and at follow up, two weeks after T1 (T2). Qualitative and quantitative methods will be used to investigate the effects of the intervention on mood, social interaction and quality of life of the PWD, sense of competence of informal carers, empathy and personal attitude of the formal carers, and quality of the relationship between the PWD, and their (in)formal carers. In addition, a process evaluation will be carried out by means of semi-structured interviews with the participating residents and (in)formal carers.

Finally, an implementation package based on the process evaluation will be developed, allowing the scaling up of the intervention to other care institutions.

**Discussion:**

Results of the trial will be available for dissemination by Spring 2023. The digital Photo-Activity is expected to promote meaningful connections between the resident with dementia, and their (in)formal carers through the facilitation of person-centered conversations.

**Trial registration:**

Netherlands Trial Register: NL9219; registered (21 January 2021); NTR (trialregister.nl)

## Background

### Introduction and rationale

Knowing the person one cares for and acknowledging their experiences and needs through genuine communication and interpersonal relationships- are essential elements of good dementia care [1]. The person-centered or person-oriented care approach stands as a contrast to the more traditional medical care model that focuses on the disease and the needs of the staff and organization, rather than the individual’s unique needs and lived experiences [1, 2]. As the symptoms and progression of dementia vary between the different types of dementia, the person, and the person’s way of dealing with the disease, it is vital that the care offered is personalized in a way that acknowledges the person with dementia’s identity and life story, especially at times when they may feel that they are losing their sense of self [3, 4].

Brooker [5] acknowledged that person-centered care may hold a variety of meanings for a number of different contexts, and while the work of Carl Rogers [6] on client-centred therapy is well-known in this area, it was Kitwood who first introduced the term person-centred approach specifically in the field of dementia care [7]. Similar work in dementia care can also be seen from Feil’s validation therapy in the United States of America [8] that provided caregivers with techniques to validate the feelings of the persons with dementia (PWD) without judging for factual accuracy. A person-centred approach is also shown in the work of Dröes [9–11] in the Netherlands, who developed the adaptation-coping model to help caregivers understand behavioural and mood symptoms as possible expressions of coping behaviour, as well as an expression of the difficulties PWD’s experience in adjusting to the cognitive, social and emotional changes in their lives. The model forms the theoretical basis for person-centered psychosocial interventions, such as the Meeting Centres Support Programme, which aim to support people with dementia and their carers in their process of adjustment to living well will dementia [12–14].

Using Kitwood’s [2–4, 7] extensive work on person-centered care, Brooker [5] noted that while having a single definition of person-centered care is not easy, there are common elements that have been identified. This model for person-centered care is known as V+I+P+S, which stands for “valuing PWD and those who care for them (V), treating PWD as individuals (I), looking at the world from the perspective of the PWD (P), and a positive social environment in which the PWD can experience relative wellbeing (S)” [4], p. 216.

In the Netherlands, an emphasis on understanding the realities of the PWD from their own perspective has led to the concept of emotion-oriented approaches [15]. First introduced by Verdult [16] in the Netherlands and Belgium as adapting care in order to fulfil the emotional needs of PWD, it was later expanded upon by Van der Kooij [17] through the concept of integrated emotion-oriented care, bringing together core elements of emotion-oriented psychosocial interventions, such as validation, reminiscence, psychomotor therapy and sensory stimulation, in daily care. Dröes [18] also emphasized that emotion-oriented care approaches were not just fulfilling emotional needs but also aimed at social needs as well as quality of life. For the purpose of this study no distinction will be made between person-centered care and emotion-oriented approaches.

In nursing homes, as well as residential and day care settings, there have been a number of controlled trials demonstrating that compared to usual care, personalized care is more effective and beneficial for PWD [19–21]. These include interventions such as the enriched opportunities programme [22], an intensive education and training program for nursing home staff on person-centered care [23], tailored nature and outdoor activities [21], personalized and comprehensive Meeting Centres Support Programme [12, 20], dementia-care mapping [19, 24], and the recently investigated *individualized* Meeting Centres Programme (iMCSP) offering people with dementia volunteer work [14]. Finnema et al. also argued for the effectiveness of emotion-oriented approaches, such as reminiscence and validation, as they focus on the unique needs and inner experiences of the PWD, and meets them where they are in the moment [15].

Considering the person’s preferences and individual wishes, providing personalized and genuine opportunities for connection, and having activities that give meaning to the person with dementia, are also believed to be essential in creating a care environment that feels more like a home rather than an institution [25, 26]. This is important because the transition of care from one’s own home to the nursing home can be a challenge, not only for the PWD, but also for the family members who may need to come to terms with letting go of the care responsibilities and entrusting their loved ones to professional carers [25, 27–29]. It was noted in one study that despite transferring the care of their loved one to the nursing home, the majority of the family informal carers still provided care, in addition to their visits [30]. In another study, it was found that family’s feelings of guilt remain stable over time after their loved one is admitted to the nursing home [31]. Nursing homes in the Netherlands are therefore working towards creating a balance between providing professional care services and creating a homely living environment for their residents through person-centered care [25]. Related to this, previous work has been done by Dröes et al. for example, in identifying the domains of quality of life that matter to PWD [32], and by Gerritsen et al. [33] to examine to what extent professional carers provided attention to these quality of life domains in their daily care. It was found that the quality of life domains ‘sense of aesthetics in the living environment’ and ‘being of use/giving meaning to life’, which were valued by PWD, received relatively little attention in the care provided by the carers [32, 33]. Knowing what is important to the individual resident, and prioritizing person-centered, psychosocial interventions in nursing homes may improve the quality of life of the residents, ensuring that even in the final stages of their life, rather than bleakly surviving day to day, residents are given a chance to thrive and to find meaning in their lived experiences [32, 34].

A type of psychosocial intervention that was found to have positive influence on the well-being of the PWD and their carers is the viewing of art on touch-screen, tablet computers [35, 36]. The intersection between technological innovations and art interventions is indeed worthy of further investigation, as, growing research on touchscreen-based interventions have shown potential in improving the psychological well-being of PWD [37], while art based interventions have been found to not only benefit PWD, but their caregivers as well [21, 38–40]. Tyack et al.’s [36] small-scale, mixed-methods exploratory study found that viewing art together on a touchscreen tablet computer via an app that they had designed, can have the potential to improve well-being of PWD and their caregivers. The art on their app consisted of images and photos that they used with permission from three London museums, as well as from collections of a painter and a photographer. The images varied from painting, decorative arts and sculptures from 16^th^ and 21^st^ century European art, objects from early Greek and Egyptian cultures, as well as rural and urban photography. It was noted that in the study, discussions around these general art images inspired spontaneous personal reminiscence and cognitive stimulation for some of the participants, without it feeling like a test of memory, unlike other traditional reminiscence therapy activities [36].

Theijsmeijer et al. [35] took the findings from Tyack et al.’s [36] study one step further in two randomized controlled pilot studies by trying to distinguish which kinds of artistic photos would have the most positive effect on mood and social interaction in persons with moderate to severe dementia living in nursing homes. The photos used in the photo interventions were compiled in a database by the artist, Laurence Aëgerter, and the photos had previously been used with PWD in nursing homes to help encourage social interaction. Groups were either shown portraits with positive facial expressions (intervention 1) versus neutral ones, or person-oriented photos (intervention 2) versus non-person-oriented photos. In the second intervention, the person-oriented photos were not the PWD’s own personal photos, rather these were general, artistic photos relating to interests they had in their youth and as young adults. Although no statistically significant differences in mood and degree of social interaction were found between the two intervention groups, possibly due to the small sample size, there was a medium to large effect size found for the intervention that utilized person-oriented photos. They seemed to have a more positive effect on speech, mood, negative behaviour and social interaction.

Theijsmeijer et al.’s [35] small scale pilot study proved to be a feasible intervention in the nursing home, and with the promising results, this intervention could provide a convenient and meaningful person-centered activity for PWD in the nursing homes. The intervention could be a non-threatening way of stimulating cognitive functioning and provide opportunities to connect and engage with their (in)formal carers by having beautiful artistic photos to talk about [35]. Generic photographs have in the past, been known to produce more in-depth personal stories from PWD compared to when they are shown personal, family photographs [41].

The current study builds upon all of the findings mentioned above, and extends on the work of Theijsmeijer et al. [35], by aiming to develop a digital, tablet version of the person-centred Photo-Activity intervention, in a larger-scale study. Digitalizing the intervention and making it available to use on a tablet computer may make it easier for staff in the nursing home to implement, as compared to having to use a physical set of photographs. In this way, new photos can easily be uploaded and accessed via an online database. An app may also have the potential for further customization of the intervention, thus making it more person-centered. This study also has the potential to address the gap identified by Hoel et al. [42] in a systematic literature review that found a lack of good quality research in the use of technological devices to support communication and meaningful social interactions between PWD and their (in)formal carers. According to the review, tablet computers that ran apps designed for reminiscence activities for PWD were the most common device used to facilitate communication between PWD and their (in)formal carer. Hoel et al. noted that most of the studies that were included in the review did not mention frequencies and duration of the interventions, and only two out of the seven studies that used a tablet computer included a control condition [42–44]. In addition, among studies in the review that used tablet computers in a nursing home [45–47], most of it involved a small number of residents with dementia [45, 46]. Despite this, most of the studies involving tablet computers and reminiscence activities that foster communication seemed to have positive effects, received acceptance, and in general, were optimistic about the benefits of progressing this line of research through large-scale, controlled studies [36, 43–48].

The digital version of the Photo-Activity has the potential to more easily scale up an intervention that can improve social health [49] and connection in the nursing home, which is proving to be even more relevant in light of the present global situation. Due to the COVID-19 pandemic nursing homes in the Netherlands and around the world have been forced to either lock-down or to restrict visits from friends and family, which is having a negative impact on the health and well-being of its residents [50, 51]. It is therefore important to not only include as much meaningful interactions as possible, that can address social isolation and loneliness [51], and can improve mood and quality of life [52], but also to harness the potential of technology in enhancing the quality of social interactions between the person with dementia and their (in)formal carers [42].

### Objectives

The current study follows the steps of the participatory action research [53] and investigates the development and design of a digital person-centered Photo-Activity intervention (Phase I) as well as its implementation and impact on the social interaction, mood and quality of life of persons with advanced dementia in nursing homes, in comparison to a control intervention in which persons with advanced dementia are offered general conversation sessions (Phase II). The aim of the Photo-Activity intervention for the PWD is to feel more acknowledged as a person, and thus experience more social connection and improvement in their mood and well-being. For the informal carers (could be family, relatives, or other close person that is not in a professional caregiver role) sharing information on the PWD’s interests and thus aiding the provision of person-centred care by the professionals in the nursing home may help the informal carers to be able to accept and better cope with the transition of their loved one’s care to the nursing home, thus enhancing their sense of competence. Finally, for the formal carers, the Photo-Activity is a means to learn about and get to know the person with dementia better, in addition to providing the formal carers tools to enable them to work in a person-centered way and contributing to a more empathic and person-centred attitude.

Based on the experience with the implementation of the intervention in the present study, an implementation toolkit for the digital photo-intervention will be developed, if the interventions proves feasible and has been evaluated positively (Phase III).

Furthermore, the current study aims to answer the following research questions:

1. What kinds of questions could be asked to the loved ones of the PWD in order to gain good insight into their personal interests?
2. How can the digital Photo-Activity intervention (app + implementation) be designed in a user-friendly manner, in a way that connects with the interests and possibilities of the person with dementia?
3. Does the Photo-Activity intervention affect the mood, social interaction and quality of life of people with dementia in nursing homes, person-centered attitude and empathy of caregivers and sense of competence of loved ones?
4. Do participants in the Photo-Activity intervention (and their loved ones/informal carers) feel more known by the formal carers, compared to the people with dementia (and their loved ones/informal carers) who do not participate in this intervention?
5. Does the Photo-Activity intervention strengthen the relationship between people with dementia, loved ones/informal carers, and formal carers?
6. What are conditions for successful implementation of the intervention at the user and organization level?
7. Which implementation materials are needed to secure and to disseminate the intervention?

## Methods/ Design

In Phase I, a user-friendly, digital photo intervention will be developed in co-creation with project group members who are experts in their respective fields (psychosocial care in dementia research, psychogeriatric care, nursing, old age psychiatry, clinical psychology, visual arts, information and communication technology), and potential users including care providers, and family/relatives of persons with dementia, and PWD themselves.

**Fig. 1:**
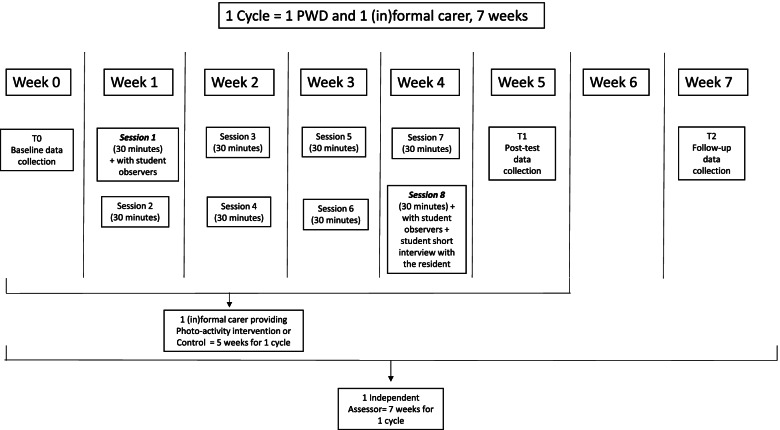
Description of one cycle involving a PWD and their (in)formal carer

In Phase II, the effectiveness of the digital Photo-Activity will be explored in a randomized controlled trial (RCT). Residents with moderate to severe dementia in nursing homes willing to join the study will be randomly assigned to participating in either the digital Photo-Activity with the (in)formal carer (experimental group), or having general conversations with their (in)formal carer (control group). Differences between the groups will be investigated with regards to their mood, social interaction and quality of life. The control group receives a general conversation activity in order to eliminate the possibility of the Photo-Activity having any effects due to residents receiving additional attention alone. Measurements of outcomes will be taken at three time-points: T0- baseline, T1- end of the 4-weeks intervention, and T2- follow-up, 2 weeks after T1 (Fig. 1).

The groups will be stratified on severity of dementia, based on a Global Deterioration Scale (GDS) [54] score of 4 to 6, the person doing the intervention (informal carer who can be a family member or relative; or a formal carer), and nursing home. Residents will be randomized after stratification. Allocation of residents with dementia and (in)formal carers to the experimental or control group will be done via manual drawing of lots by the researchers, and (in)formal carers are informed of the assignments afterwards.

In addition, effects of the experimental intervention versus the control intervention in informal carers will be investigated regarding their feelings of competence. Effects on the (in)formal carers who provide the Photo-Activity intervention (experimental group) or the general conversation sessions (control group) with the residents, are compared regarding attitude and empathy in both groups.

During the trial period, a process evaluation will also be conducted where residents and their (in)formal carers at the end of the 4-week intervention will be asked about their experiences with the implementation of the Photo-Activity intervention, in order to gain insight into factors that may have influenced the study outcomes, such as the user-friendliness and usability of the Photo-Activity app, the frequency of use, and facilitating and impeding factors towards successfully implementing the Photo-Activity intervention. Residents who participated in the general conversation activity and the (in)formal carers who delivered this activity will also be asked about their experiences.

In Phase III, based on the results of the process evaluation, an implementation toolkit will be developed as described in the Objectives.

### Study setting and participants

The study will be conducted in 14 nursing home wards that are part of 2 large care organizations in the Netherlands (in the Amsterdam and Haarlem region). PWD in these participating nursing home wards who meet the inclusion and exclusion criteria (regardless of gender, age, socioeconomic status, and ethnicity), along with their legal guardians, will receive a written invitation from the nursing home with a consent form for participation and information about the study.

Based on the pilot study with the Photo-Activity, in which large positive effect sizes were found for social interaction (d= 0.86) and reduced negative behaviour (*d*=0.85) using the INTERACT scale [55] during the intervention [35], a power-analysis (power 0.80, α=0.05, *d*=0.8) and expected 10% dropout in 6 weeks, for this explorative study 45 people with dementia are needed per group (total of 90) to find statistically significant large effects. The researchers will promote the project in the care organizations and work with the research coordinators, ward managers and team leaders in order to meet the required recruitment target.

### Eligibility criteria

#### Inclusion criteria

Residents with dementia (Alzheimer’s disease, vascular dementia, Mixed Alzheimer’s-vascular dementia, front-temporal dementia, Lewy Body disease, or others) whose score range from 4-6 (moderate to severe dementia) on the GDS [54], and have been admitted to the nursing home for at least 1 month are eligible for the study. Those who give their consent and who agree to participate will be included, and after stratification (severity of dementia; intervention provider; and nursing home), will be randomly assigned to an experimental or control group. Formal carers will also be randomly assigned to deliver the experimental or control activity.

For the informal carers, they will be assigned to the same activity where their loved one is randomly assigned to, and there are no specific inclusion criteria, except that they are willing to participate in the study themselves as well as to provide information on the interests of their loved one. If they would like to be involved in carrying out the Photo-Activity or control intervention for their loved one, they additionally must be willing to participate in a short training to deliver the intervention and be able to commit to providing the intervention for 2 half hour sessions a week, for a period of 1 month.

The formal carers of the included PWD’s are invited and trained to deliver the experimental/control intervention and to be a part of the study after providing informed consent. The recruitment of formal carers will focus on those who work enough hours to fulfill the criteria of delivering the experimental or control activity twice a week for 4 weeks. Information about characteristics of the formal carers delivering the intervention (work function, training level, experience, working hours, etc.) will be collected.

For formal carers assigned to be Independent Assessors, i.e. not involved in the interventions, recruitment criteria are that they are present in the ward for more than 2 days a week, and have regular contact with the residents. The Independent Assessors are asked to complete outcome measures about the residents. Because no information is collected on the Independent Assessors themselves, they are not asked to fill out the consent forms.

#### Exclusion criteria

People with mild (GDS 3) or very severe dementia (GDS >6) and those who have severe loss of hearing and/or vision will be excluded from the study.

#### Blinding (masking)

In order to prevent bias and to ensure that the intervention conditions are met (and not mixed up), the care providers from the 14 nursing home wards involved in the study will be informed of which persons in the ward are assigned to the experimental or control groups. Only (in)formal carers of the residents in the experimental group however, will be informed in detail about the Photo-Activity intervention during the study. Professional carers who are not the primary care provider (thus not providing either the experimental or control activity) will be invited to participate as Independent Assessors to fill in the outcome measures. They will not be informed on which residents will be offered the experimental or control intervention. Due to the small teams working in each ward, especially those in small-scale living arrangements, it might not always be possible to blind the Independent Assessors from knowing which intervention sessions the residents are assigned to. However, because the Independent Assessors are not present during the experimental or control sessions or trainings, and they do not provide the interventions themselves, the risk of bias is limited.

## Interventions and procedures

### Photo-activity intervention (experimental group)

#### Training

The (in)formal carers assigned to the experimental group, will be informed about the background, method, and application of the Photo-Activity intervention app, by a member of the research team. A short video from the artist Laurence Aëgerter, who originally developed the intervention, will also be shown.

A 75-minute training session on how to hold conversations around the artistic photographs with the residents will be given by a senior member of the research team. The training will include basic interview techniques that foster rapport, like asking open questions instead of closed ones, reflective listening and summarizing. The (in)formal carers in the experimental group will also be trained to use ‘person-enhancing communication strategies’ such as offering comfort (in the form of warmth, security, and conversing in a relaxed pace), supporting identity (through respect and acceptance), and offering opportunities for attachment (recognition, affirmation, sincerity) as opposed to ‘predatory communication strategies’ such as withholding comfort (being intimidating, abstaining, speeding up the conversation), not supporting identity (childish, labelling, belittling), and not allowing oneself to be attached (accusing, deceiving, not acknowledging) [4]. Finally, they will also be instructed to avoid questions that would seem to ‘test’ the residents about their interests or to recognize certain photos; rather any response from the residents is acceptable i.e. there is no right or wrong. The (in)formal carers are informed that all the information on ‘person enhancing communication strategies’ can also be found in the Photo-Activity app.

(In)formal carers participating in the training will be given their own unique code to access the app, besides a unique code they receive for each resident to which they provide the Photo-Activity. They will be invited to spend time on the app in order to familiarize themselves with the functions, i.e.: selecting a series of photos based on the interests profile of the resident with dementia, saving photos in the ‘conversation basket’, showing the photo series in full screen presentation mode, selecting favorite photos, or taking notes about photos that elicited the most/least reactions, or were more/less appreciated by the resident.

#### Intervention

The resident and their trained (in)formal carer will meet for 30 minute sessions, two times a week, for 4 weeks to talk about artistic photos on the app which they will view together using a tablet computer. The photos will be selected by the (in)formal carer delivering the Photo-Activity, based on a personal interest profile in which the interests of the resident, as inventoried in a pre-interview with them and/or their (in)formal carers, are described. Once 15-20 photos reflecting the interests of the resident are chosen from the app, the (in)formal carer spends half an hour with the resident in a quiet place, offering them something to drink while looking through the photos on the tablet. There is no set time for viewing each photo, and there is no requirement that the whole set of 15-20 photos should be viewed. They can spend as much time as they like viewing one photo for as long as reactions or stories are evoked from the resident.

### General conversation activity (control group)

#### Training

The (in)formal carers in the control group, will receive a short 45-minute training by a senior member of the research team to inform them about what the research project entails. They will be instructed to hold general conversations on random topics with the resident. They can freely choose the topic or address the topics that the resident brings up. No photos will be shown during the general conversation activity. The (in)formal carers in the control group will also be trained in engaging the resident in conversation using basic interview techniques for rapport building (open and closed questions, reflective listening, summarizing). They do not receive training in person-enhancing communication strategies.

#### Intervention

For the control group, the (in)formal carers will also have 30 minute sessions with the resident, two times a week for four weeks, in a quiet place within the nursing home. They will offer the resident something to drink, and spend the 30 minutes having a general conversation.

### Procedure for student observers

Research interns and nursing students will receive a 60-minute training session from a member of the research team in observing the behaviour of the resident during the experimental and control conditions, using the INTERACT [56]. The INTERACT scale has 22 items with 5 answer options ranging from ‘Not at all’ to ‘Nearly all the time’, and is used to rate the mood, speech, relation to another person or to the environment, need for prompting, and stimulation level of a person with dementia during a therapy session [57], as well as any negative interactions like reluctance, complaining or aggression [58]. The students will also rehearse how they can give constructive feedback to the (in)formal carers carrying out the intervention regarding communication styles and techniques when communicating with the resident.

## Outcome measures and products

For Phase I of the study (Design and development of the Photo-Activity intervention app), data will be collected in writing from user feedback meeting rounds and user testing of prototypes. Feedback, comments, and insights from the user groups ((in)formal carers, PWD and researchers) will be collected per meeting and reported anonymously. At the end of the development phase, the System Usability Scale (SUS), a short 10-item questionnaire that assesses perceived usability [59], will be given to the (in)formal carers- who were involved in the app development to evaluate its usefulness and user-friendliness. Observations of the persons with dementia themselves during the user tests will be conducted by trained student observers with the INTERACT observation scale [56] (see Phase II outcomes).

Data from Phase II (Implementation and assessment of impact of the Photo-Activity intervention) will be partly quantitative and partly qualitative. Data on background characteristics based on The Older Persons and Informal Caregivers Survey Minimum Data Set or TOPICS-MDS [60]; -such as age, gender, ethnicity, civil/marital status, education level, children, hours of paid or volunteer work, work function will be collected from the PWD and their (in)formal carers. In addition, the following primary and secondary outcomes will also be collected:

### Quantitative measures

#### Primary outcomes

For the residents, positive emotions and social interaction with their (in)formal caregiver during the activity session are assessed by trained students with the INTERACT observation scale [56] during the first and last intervention session.

Quality of life is evaluated by formal carers who are assigned the role of Independent Assessors, with the QUALIDEM [61] based on their observations of the behaviour of the resident in the previous week at T0, T1, and T2. These formal carers are trained in the use of the instrument by a member of the research team. The QUALIDEM was developed specifically to measure quality of life of residents with dementia living in nursing homes, as rated by the formal carers. It is made up of 37 items that describe behaviour that can be observed by the carer. Carers can choose from the answer options of ‘never, seldom, sometimes, and often [62]. The QUALIDEM has been found to have sufficient reliability and validity, and is an easy questionnaire to administer [61, 62].

#### Secondary outcomes

For the residents, their mood will be assessed with the one item self-reported five-point visual analogue scale known as the Smiley Face Expression Scale (SFAS) [63] during the first and last intervention week (2 sessions per week). The faces in the SFAS corresponds to moods that reflect a range from ‘very unhappy’ to ‘very happy’, and the SFAS has been previously used in other studies to evaluate mood of PWD [63–65]. There seems to be no previous studies that have established the validity of the SFAS, but it was found to be an effective measurement of PWD in an art museum intervention [63], and in general, similar visual analogue scales that measure mood of PWD have shown good validity [64, 66].

The Independent Assessor’s assessment of mood and behavioural symptoms of the resident will be evaluated with the brief Neuropsychiatric Inventory (NPI-Q), for assessing psychopathology in dementia [67] at T0, T1 and T2. The NPI-Q was found to be a reliable and valid assessment of neuropsychiatric symptoms, covering 12 symptom domains [67, 68]. The carer rates the frequency (ranging from 1-4) and severity (ranging from 1-3) of symptoms, and also rates their level of distress for every symptom present, from 0 (not distressing at all) to 5 (extremely distressing). The care provider answering the NPI-Q is the same one who fills out the QUALIDEM [61].

Person-centered attitude of the (in)formal care providers involved in the experimental or control conditions with the resident is examined with the Approaches to Dementia Questionnaire (ADQ) [69], which has 19 statements about resident and how they are cared for, with a five-point Likert scale ranging from ‘strongly agree’ to ‘strongly disagree’. The ADQ has been found to have good validity and reliability [69–71].

Empathy will be measured using the Interpersonal Reactivity Index (IRI), which has 28 items measuring global empathy, with subscales on perspective taking, fantasy, empathic concern, and personal distress. It can be answered on a 5-point Likert scale ranging from ‘does not describe me well’ to ‘describes me very well’ [72]. The IRI has been found to be valid and reliable [73].

Both the ADQ [69] and the IRI [72] will be completed on T0 and T1 by the (in)formal care providers in the experimental and control groups. (In)formal carers in the experimental condition will additionally be asked to complete the System Usability Scale (SUS) [59] at T1. SUS is a well-known brief scale for measuring perceived usability, and has been found to have excellent reliability and concurrent validity [74].

For the informal carers, their feelings of competence in dealing with the PWD is evaluated using the Short Sense of Competence Questionnaire (SSCQ) [75] during T0, T1 and T2. The SSCQ, which assesses issues experienced by the informal carer, has 7 items that can be answered on a 5-point Likert scale ranging from ‘completely agree’ to ‘completely disagree’. It has satisfactory validity and reliability [76].

For the duration of Phase II, intervention compliance will be monitored by asking (in)formal carers to record the dates, times, and duration of their sessions with the residents in both experimental and control group, along with life events that may impact the residents or (in)formal carers (such as illness, hospitalization, or death of loved ones) and other reasons for drop-out.

A summary of outcome measures and timeframe for completion is shown on Table 1.

**Table 1:** Quantitative outcome measures and timeframe for completion

	T0	During Intervention Period	T1	T2
**Primary outcomes**				
Student observer/ PWD		INTERACT Observation Scale (Session 1 and 8)		
Independent assessor	QUALIDEM		QUALIDEM	QUALIDEM
**Secondary outcomes**				
PWD		SFAS (1^st^ and 4^th^ intervention week)		
Independent assessor	NPI-Q		NPI-Q	NPI-Q
(In)formal carer (experimental/control)	TOPICS-MDS ADQ IRI		ADQ IRI	
(In)formal carer (experimental only)			SUS	
Informal carer only	TOPICS-MDS SSCQ		SSCQ	SSCQ

The QUALIDEM specifically measures quality of life of residents with dementia living in nursing homes, as rated by formal carers. The brief Neuropsychiatric Inventory (NPI-Q) assesses mood and behaviour symptoms of the residents. The Older Persons and Informal Caregivers Survey (TOPICS-MDS) is for collecting background characteristics of residents and their (in)formal carers. The Approaches to Dementia Questionnaire (ADQ) measures person-centered attitude of (in)formal carers who provide the intervention or the control activity. The Interpersonal Reactivity Index (IRI) measures empathy. The Short Sense of Competence Questionnaire (SSCQ) measures feelings of competence of the informal carer in dealing with the PWD. The INTERACT is an observation scale used to rate the behaviour and interactions of the PWD with their (in)formal carer. The Smiley Face Expression Scale (SFAS) is a self-reported mood assessment for the PWD. The System Usability Scale (SUS) measures perceived usability

### Qualitative measures

To investigate whether informal carers feel that their role and their relationship to the person with dementia is known and acknowledged in the nursing home, the following questions in focus groups and semi-structured interviews will be asked on T0, T1, and T2:

1. To what extent do you feel that you are known as a family/informal carer in the nursing home?
2. How satisfied are you with the stay of your loved one in the nursing home?
3. To what extent do you think you (or your loved one) are acknowledged in the nursing home?

To investigate the extent to which the intervention is able to strengthen relationships between the resident and their (in)formal carers, the (in)formal carers who deliver the Photo-Activity intervention or the control activity will be asked the following in focus groups and semi-structured interviews at T1:

1. To what extent did you get to know the resident and his or her family member better as a result of the Photo-Activity intervention/ general conversation activity?

Finally, a process evaluation, via semi-structured interviews with the (in)formal carers and residents, will be done to evaluate the experiences with the Photo-Activity intervention or the control activity. To inventory the factors potentially influencing the outcomes of the study, the researchers will use a pre-determined list of questions based on the Medical Research Council (MRC) guidance for process evaluation of complex interventions [77], where contextual and implementation factors and impact mechanisms (learnability, user-friendliness, usability and adoption) are explored as factors that may have an influence on the intervention outcomes.

Analytics data are also collected from the Photo-Activity web-app (called the *Fotoscope),* via Google Analytics to be able to assess how the web-app was used. Users are given unique usernames that are distinct from their participant code in the study, so that data from Google Analytics is not identifiable, except for members of the main research team who holds the key to the codes.

Two focus groups, based on purposive sampling, will also be held as part of the process evaluation of Phase II, involving residents with a diverse background in terms of age, gender, cultural background, and severity of dementia, together with the residents’ loved ones, (in)formal carers, and the ward managers (stakeholders). Three to eight participants per group will be formed in order to give the participants more chances to contribute to the conversation [78]. The focus groups, led by a moderator and observer, will be conducted face-to-face, or via online video-calls, depending on the current government COVID-19 restrictions.

In Phase III, the implementation toolkit will be compiled to promote sustainable implementation, dissemination, scaling up and assurance of knowledge. The toolkit will consist of the Photo-Activity app, and a digital implementation guide (fact sheet) for the management of care institutions, validated by a panel of care providers, PWD, family members, teachers (MBO and HBO), managers and researchers.

### Data collection procedure

Recruitment period and data collection is currently on-going, starting from February 2021 to June 2022.

### Quantitative data

One staff-member from the ward is assigned the role of the *coordinator*, who becomes the primary contact of the researchers in terms of recruiting residents and their families, and collecting informed consents. The coordinator sends out the project invitation along with the informed consent forms to the families of the residents. Once the coordinator gains the informed consent of the resident (where possible) and their legal representative, the researchers then contact and start collection of demographics and baseline information via sending online questionnaires to the (in)formal carers. The online questionnaires include outcome measures mentioned above. All questionnaires are sent at T0, T1 and T2, except for the demographic/background questionnaires which are only sent at T0, and for the questionnaires specifically for (in)formal carers delivering the intervention or control activity which are sent at T0 and T1 only.

Medical information such as type of dementia, severity of dementia, and information on medication related to dementia are collected by the researcher from the coordinator at T0. Medication information is again collected at T1 and T2.

The (in)formal *carers delivering the interventions* (either the Photo-Activity or General Conversation activity) are asked to keep a record of session details, including date and time, duration, and reason if a session is missed. The (in)formal carers also collect information about the resident’s mood before and after a session, using the SFAS [79]. This is done at the first and last week of the intervention period (in total, data on residents’ mood is collected 4x2 times).

The formal carers acting as *Independent Assessors* receive training on how to assess the resident, and based on their observations, to fill out the QUALIDEM [61], the NPI-Q [67], and to ask the residents a couple of questions from TOPICS-MDS [60] about their health and life satisfaction, and questions about feeling known in the nursing home, and levels of satisfaction with their stay in the nursing home. Independent Assessors are also asked to note down any significant events in the resident’s life (for example, when a resident becomes ill), during the intervention period. These data are collected through online questionnaires and are done at T0, T1 and T2.

Data on residents’ positive emotions and social interactions with their (in)formal carers will be collected by trained students or interns using the INTERACT scale [56]. They will be present to observe the first and last Photo-Activity or General Conversation activity via online video-calls, keeping in line with the Covid-19 restrictions.

### Qualitative data

#### Phase II

At the end of the four-week intervention period, a process evaluation is conducted by asking the residents and their (in)formal carers about their experiences in being part of the Photo-Activity or the General Conversation Activity, as described above. The residents are interviewed by the student observer during their last session, via online video-call. The (in)formal carers are given the option of completing the list of questions on their own time, or answering the questions via phone-conversation with the student observer.

#### Phase III

Two focus groups, one from each participating care organization, will also be held to validate the implementation toolkit, as described above. Approximately 1.5 hours in length, the discussions will involve the functionality and design of the draft toolkit, keeping in mind ways to adapt the mode of instruction to different target groups.

### Data management and monitoring

The researcher contacts (in)formal carers at the start of the project (after gaining informed consent), and follows-up with data collection during the entire period. The researcher encourages the (in)formal carers to ask questions and to contact the research team for any comments or feedback. Upon recruitment, residents and their (in)formal carers are informed that personal data are collected and will be treated as confidential, i.e. all data will be anonymized and linked to a unique participant code and saved in a secured and privacy protected database. The key to the code that links to their personal data is only accessible to the research team and will be destroyed after the end of the project. Usernames for residents and (in)formal carers to access the digital Photo-Activity app are given another separate and unique, random code, of which the key linking the app usernames to the participant personal data codes is again only accessible by the members of the research team.

The research data cannot be traced back to the individuals in case of publication of research results in any journal or publication. Signing the consent forms will imply that participants consent to the researchers collecting, storing, and accessing their personal data, and that these data will be kept for 10 years after the end of the study period. Participants are informed that they are free to withdraw themselves and their personal data from the study at any time. Participants are also asked if they consent to their coded data being added to a national database that will be made available for new studies.

Personal and quantitative trial data will be collected and stored online, via Castor Electronic Data Capture (Castor EDC), a cloud-based clinical management platform [80], that complies with the Good Clinical Practice (GCP) laws and regulations. Data in Castor is regularly backed-up, retrievable, and trackable with an audit trail in Castor EDC’s secure servers based in the European Union. Access to the database is protected by a password and the use of two-factor authentication. Each Castor user is assigned a role that determines the level of access they have to the data.

For data that needs to be collected in paper form and sent via email from the nursing homes, researchers will request the nursing home staff to send these documents via Zivver [81], which is a platform that allows secure sending of sensitive information and files via email. Once received by the researcher, names of participants are removed and the files are coded, saved, and stored in the secure, password-protected, digital workspace used by the Department of Psychiatry within the mental health organization GGZ inGeest.

Original and printed documents are also anonymized, coded, and stored securely in a locked office. Informed consent forms are stored in a locked drawer to which only the research team has access. Any copies containing personal information are disposed of via a secure and confidential document disposal service.

The researcher monitors and keeps track of the progress of data collection, following-up missing data or addressing data discrepancies, via the reminder function in Castor, email communication or follow-up phone calls with the participants.

The project has been assessed scientifically and is embedded within the Amsterdam Public Health (APH) research institute, which holds regular sample audits, therefore a data monitoring committee is not needed. The researcher ensures that the project is in line with the guidelines set by the APH research institute [82].

A Data Management Plan for the collection, management, analysis and storage of the data will be created in line with Open Science, and based on the principles of FAIR data (Findable, Accessible, Interoperable, and Reusable). Data from the survey will be made available to the TOPICS-MDS, a public data repository in the Netherlands [83].

## Analysis

### Quantitative data

Analysis for quantitative data will be done using SPSS for Windows. Participant characteristics are analyzed with descriptive statistics and differences are tested with parametric or non-parametric tests dependent on type of data. Multilevel analysis (MLA) is performed to investigate whether the averages on continuous outcome measures at T1 and T2 differ between experimental and control groups. Baseline measurements (T0), severity of dementia and potential confounders are included in the analyses as covariates. The MLA takes into account the clustering of people with dementia and caregivers within nursing wards. The same procedure is used for dichotomous data, but the analyses are then performed with General Estimations Equations. Both intention-to-treat and ‘per protocol’ analyses will be done.

### Qualitative data

Descriptive statistics via SPSS for Windows will be used to analyze the characteristics of the interviewees, care providers and other stakeholders. Audio recordings from focus groups will be transcribed. The transcriptions and meeting notes of the focus groups and interviews with (in)formal carers, and stakeholders will be analyzed thematically using deductive and inductive methods [84]. The software Atlas.ti will be used.

### Ancillary and post-trial care

If the participating nursing homes view the Photo-Activity intervention as a positive addition to their services, then the (in)formal carers will be given free access to the web-app after the study period is completed, to avoid data of active and non-active study participants being collected in Google Analytics. However, for residents in the Photo-Activity, they will still be able to use the web-app after follow-up at T2, because the researchers will have a record of the Photo-Activity session times that they can match to Google Analytics.

Training on the use of the web-app and the Photo-Activity intervention will also be made available to nursing staff of the participating nursing homes who are interested in the Photo-Activity, but provided the control intervention or were not involved in providing the interventions. If the intervention proves to be effective, an implementation toolkit will be developed to promote further implementation in the participating nursing homes, as well as dissemination to other nursing homes across the country.

### Dissemination policy

Results of the current study are expected to be available in Spring 2023. The project is linked to the Marie Curie Innovative Training Network DISTINCT, in which 15 early-stage researchers at 10 universities in 7 countries are researching the use of technology to promote social health in people with dementia and their carers. This has given the project international attention right from the start. Via the DISTINCT website and project partner Alzheimer Europe, results are easily disseminated internationally. Research results will be published in open-access and national/international scientific professional, and public (online) journals for nursing, gerontology, psychiatry and technology. Because the project was classified by the Medical Ethics Review Committee of VUmc as non-medical research, any amendments made to the protocol will be noted and written in the final reporting of the study.

## Discussion

With the accessibility and convenience of tablet-computers, the digital, person-centered Photo-Activity intervention is expected to be a simple and user-friendly activity for formal and informal carers alike to provide to PWD living in nursing homes and other residential care settings. The web app can be accessed via internet browsers on tablets or mobile phones, and thus can be easily used and brought to any space (living rooms or bedrooms) in the nursing home. It is expected that the Photo-Activity intervention will enable the PWD to connect meaningfully with their (in)formal carers through a social activity that acknowledges their personal interests and respects their personhood. At the same time, this activity provides a playful way for (in)formal carers to engage with the residents, as often, they are uncertain about how they can have conversations with residents who are in an advanced stage of dementia.

Ultimately, it is expected that the resident, through feeling seen and acknowledged as a person, will experience improved mood and wellbeing as a result of the digital Photo-Activity intervention. It is expected that their informal carers will be more accepting of the transferring of care responsibilities to the nursing home when experiencing the person-centered care approach and improved relationship with their loved one’s care provider. It is expected that the formal carers will be able to use the digital photo intervention as a tool to help them work in a more person-centered way, and allow them to develop a deeper personal relationship and more empathy for the residents. The implementation toolkit provided by the project will help to implement and scale up the intervention in other care and home situations.

Finally, as an intervention aiming to harness the potential of technology, this study can provide insight into the use of technological devices to support and promote person-centered care for the improvement of the social and mental health of residents with dementia in long term care facilities.

## Data Availability

Not applicable.

## Abbreviations

ADQ: Approaches to Dementia Questionnaire
APH: Amsterdam Public Health research institute
Atlas.ti: Software package for qualitative data analyses and mixed methods research
Castor EDC: Castor Electronic Data Capture platform
DISTINCT: Dementia: Intersectorial Strategy for Training and Innovation Network for Current Technology
Foundation for Support VCVGZ: In Dutch - Stichting tot Steun VCVGZ (Vereniging voor Christelijke Verzorging van Geestes en Zenuwzieken), a charitable foundation
HBO: in Dutch- *Hoger Beroeps Onderwijs;* or University of applied sciences
INTERACT: Behaviour observation scale for social interaction
ITN: Innovative Training Network
GCP: Good Clinical Practice
GDS: Global Deterioration Scale
GGZ inGeest: Regional Dutch mental health service in Amsterdam
MBO: in Dutch- *Middelbaar Beroeps Onderwijs*; secondary vocational education
MRC: Medical Research Council
Non-WMO: The Medical Research Involving Human Subjects Act (WMO) does not apply to the study
NPI-Q: Neuropsychiatric inventory questionnaire
PWD: Persons with dementia
QUALIDEM: Dementia-specific, quality of life instrument
RCOAK: In Dutch- *Roomsch Catholijk Oude Armen Kantoor;* a charitable Catholic Foundation
RCT: Randomized controlled trial
SPSS: Statistical Package for the Social Sciences
SSCQ: Short Sense of Competence Questionnaire
T0: Baseline
T1: Four weeks after the start of the intervention period
T2: Two week follow-up after T1
TOPICS-MDS: The Older Persons and Informal Caregivers Survey Minimum Data Set
V+I+P+S: Valuing people with dementia and those who care for them
VUmc: VU University Medical Centre
